# Establishing tropical medicine as a recognized medical specialty or strengthening its training within infectious diseases residency programs in Colombia: an urgent need

**DOI:** 10.1590/0037-8682-0527-2025

**Published:** 2026-03-30

**Authors:** Carlos Ramiro Silva-Ramos, Carlos Arturo Álvarez-Moreno, Alfonso J. Rodriguez-Morales, Álvaro A. Faccini-Martínez

**Affiliations:** 1 Pontificia Universidad Javeriana, Facultad de Ciencias, Departamento de Microbiología, Grupo de Enfermedades Infecciosas, Bogotá, Colombia.; 2 University of Texas Medical Branch, Department of Pathology, Galveston, Texas, USA.; 3 Universidad Distrital Francisco José de Caldas, Facultad de Ciencias de la Salud, Programa de Enfermería, Bogotá, Colombia.; 4 Clínica Universitaria Colombia, Clínica Colsanitas, Bogotá, Colombia.; 5 Universidad Nacional de Colombia, Facultad de Medicina, Bogotá, Colombia.; 6 Universidad Científica del Sur, Faculty of Health Sciences, Lima, Perú.; 7 Fundación Universitaria Autónoma de las Américas, Institución Universitaria Visión de las Américas, Facultad de Medicina, Grupo de Investigación Biomedicina, Pereira, Colombia.; 8 Hospital Militar Central, Servicio de Infectología, Bogotá, Colombia.; 9 Universidad Militar Nueva Granada, Facultad de Medicina, Bogotá, Colombia.

**Keywords:** Tropical medicine, Tropical diseases, Colombia, Clinics, Medical education

## Abstract

Colombia is entirely located within the tropical latitudinal zone and is characterized by extensive biodiversity, complex ecosystems, and socio-environmental inequities that contribute to the sustained persistence of a wide range of tropical diseases. Despite the high prevalence and public health impact of these conditions, tropical medicine is not currently recognized as a clinical specialty in Colombia, and its inclusion within infectious diseases residency programs remains limited, fragmented, and primarily hospital-centered. As a result, future specialists may complete their training with insufficient clinical experience in endemic tropical diseases, particularly those affecting rural and marginalized populations. Although research-oriented postgraduate programs in tropical medicine exist, clinical training and direct patient management are largely absent. In contrast, several countries in Europe, North America, and Latin America have adopted specialized educational models that integrate fieldwork, interdisciplinary curricula, and strong clinical components to strengthen expertise in tropical medicine. To reduce persistent health inequities and enhance national preparedness for emerging tropical threats, Colombia urgently needs to strengthen tropical medicine training, either by expanding and standardizing content within existing infectious diseases residency programs or by establishing a dedicated clinical postgraduate specialty. Strengthening academic and clinical capacity in this field would improve patient care, promote applied research, and support evidence-based strategies for managing tropical diseases nationwide.

## INTRODUCTION

Tropical medicine is an interdisciplinary field that aims to address the complex epidemiological challenges posed by diseases endemic to tropical and subtropical regions^1^. Colombia is entirely located within the intertropical zone and has one of the highest levels of biodiversity worldwide; however, pronounced altitudinal gradients give rise to a wide range of ecosystems, including extensive lowland tropical environments that facilitate the circulation and persistence of a broad spectrum of tropical diseases^2^ ^,^ ^3^. Despite the high relevance of these diseases, tropical medicine is not yet recognized as an independent clinical specialty, and its inclusion within infectious diseases residency programs remains limited and fragmented, leaving substantial gaps in clinical training for tropical diseases that predominantly affect rural populations. The lack of structured training in Colombia underscores the urgent need to establish clinical tropical medicine programs, either by enhancing existing residency curricula or by creating a dedicated postgraduate specialty. This manuscript aims to provide a comprehensive overview of the current state of tropical medicine training in Colombia, highlight existing gaps in clinical programs, and propose potential strategies to strengthen the country’s capacity to manage tropical diseases.

## TROPICAL MEDICINE

Tropical medicine is an interdisciplinary field that encompasses the study and management of infectious and non-infectious diseases that predominantly occur in tropical and subtropical regions worldwide^1^. These areas are often characterized by persistent poverty, poor sanitation, fragile health infrastructure, limited access to timely and equitable healthcare services, and other social and environmental factors that collectively contribute to the emergence, persistence, and spread of these diseases^1^. The field addresses a broad spectrum of infectious diseases of major global relevance because of the significant morbidity and mortality they cause, including malaria, dengue, Chagas disease, leishmaniasis, and others^1^ ^,^ ^4^. Beyond infectious diseases, tropical medicine also encompasses noncommunicable conditions such as malnutrition and envenomation by venomous animals, which, although less frequent, have a substantial impact on the health and quality of life of these vulnerable populations^5^ ^,^ ^6^.

Historically, tropical medicine emerged in the latter half of the nineteenth century as a response to the health challenges encountered by European colonizers and military personnel operating in tropical territories that were perceived at the time as exotic, inhospitable, and largely unfamiliar to these populations, who were exposed to multiple infectious diseases for which there was no previous experience in diagnosis, treatment, or prevention^7^ ^,^ ^8^. Therefore, European physicians were required to confront unprecedented medical challenges involving unfamiliar pathogens, vectors, and epidemiological contexts in tropical regions. The work of the Scottish physician Sir Patrick Manson (1844-1922) marked a turning point, transforming these challenges into a new scientific discipline and leading to his recognition as the “father of tropical medicine”^9^. After completing his medical training at the University of Aberdeen, Manson practiced extensively in several regions of Asia, particularly in China, Hong Kong, and Taiwan^9^. During his clinical practice, he made pioneering observations on the relationships between hematophagous arthropods and parasitic diseases, laying the foundation for the modern understanding of vector-borne transmission^9^ ^,^ ^10^. Manson’s research demonstrated that certain mosquitoes served as essential vectors of the parasite *Wuchereria bancrofti*, the causative agent of lymphatic filariasis, representing a major milestone in understanding parasitic disease transmission^11^.

Based on these discoveries, Manson proposed the mosquito-malaria theory, which later inspired studies that experimentally confirmed the role of mosquitoes in the transmission of *Plasmodium* ^12^. He also discovered several parasitic species, including *Schistosoma mansoni*, further consolidating his position as a key figure in modern parasitology^11^. Manson’s work not only established tropical medicine as an academic and clinical discipline but also laid the conceptual foundations for disease control strategies in endemic regions by providing a scientific framework to understand transmission dynamics and guide preventive measures^9^ ^,^ ^10^. Upon returning to London, he promoted the founding of the London School of Hygiene and Tropical Medicine in 1899, a pioneering institution that has trained generations of researchers and practitioners in tropical diseases. He later became the first president of the Royal Society of Tropical Medicine and Hygiene in 1907^9^.

## COLOMBIA AS A TROPICAL COUNTRY

Colombia is located in the Neotropical region of South America, between the Caribbean Sea and the Pacific Ocean. It is crossed by the equator, placing its entire territory within the intertropical zone between the Tropics of Cancer and Capricorn^13^. Colombia’s complex topography and pronounced altitudinal gradients give rise to wide climatic diversity, primarily determined by altitude and the presence of distinct thermal zones ranging from warm coastal lowlands to cold high-mountain ecosystems. Approximately 80% of the national territory consists of lowland tropical ecosystems^3^. Such environmental heterogeneity supports a remarkable variety of ecosystems, from tropical rainforests and savannas to arid moors and mountains, making Colombia one of the most biologically diverse countries in the world^3^.

This biodiversity is reflected in the abundance of plant and vertebrate species, many of which serve as natural reservoirs for a wide range of infectious agents, including several rodent, bat, and marsupial species^14^ ^,^ ^15^. Similarly, many invertebrates act as vectors of multiple pathogens, particularly species of the genera *Anopheles*, *Aedes*, and *Lutzomyia* ^16^ ^,^ ^17^.

In recent years, Colombia has gained prominence as a major tourist destination in South America and the Caribbean, attracting international visitors primarily because of its exceptional biodiversity^18^. This increasing human mobility and the growth of ecotourism promote closer interactions among humans, wildlife, and vectors, which may facilitate outbreaks and the spread of endemic tropical pathogens and are also relevant to travel medicine^19^.

Beyond natural determinants, numerous social and economic factors further influence the eco-epidemiology of tropical diseases in Colombia. Rapid and unplanned urbanization, deforestation, mining, and agricultural expansion are among the main anthropogenic activities that disrupt ecological balance and promote the proliferation of arthropod vectors and the emergence of enzootic pathogens^18^ ^,^ ^20^ ^,^ ^21^. Furthermore, despite Colombia's recent economic growth, social and structural inequities persist, including poverty, limited access to basic public services, and forced migration and displacement related to the long-standing internal armed conflict. These conditions continue to affect large rural and peri-urban populations and contribute to vulnerability to a broad spectrum of tropical diseases, primarily infectious diseases^22^ ^,^ ^23^.

Overall, Colombia represents a setting in which multiple environmental, biological, economic, and social determinants converge, favoring the circulation and persistence of tropical diseases^21^. Consequently, tropical medicine has become a cornerstone medical field for public health in the country, not only for its role in diagnosis, management, and control but also as a strategic area for scientific research, epidemiological surveillance, and the development of evidence-based policies aimed at mitigating the impact of tropical diseases on both local populations and international travelers.

## AN OVERVIEW OF TROPICAL DISEASES IN COLOMBIA

A wide range of tropical diseases occurs in Colombia, many of which are of significant public health relevance. These include both notifiable diseases and emerging infections whose contribution to the overall disease burden remains only partially understood^2^. Among the most representative are malaria, dengue, leishmaniasis, and Chagas disease, all characterized by persistent transmission and the presence of vectors highly adapted to local ecosystems^24^ ^,^ ^25^. Additionally, infections such as rabies remain a major zoonotic threat in certain regions because of inadequate control of domestic and wild reservoirs. Simultaneously, leptospirosis and several neglected zoonoses have been recognized as significant causes of acute undifferentiated febrile illness (AUFI), particularly in rural and peri-urban populations^26^ ^,^ ^27^.

Malaria remains highly prevalent in rural tropical areas, where environmental conditions, the presence of competent vectors, and limited access to health services favor its persistence and recurrent outbreaks^28^ ^,^ ^29^. Similarly, dengue is hyperendemic in the country’s major tropical cities, a pattern sustained by the widespread distribution of *Aedes aegypti* and *Aedes albopictus*, unplanned urbanization, and social and environmental factors that facilitate virus circulation^30^ ^,^ ^31^. In addition, emerging arboviruses such as Chikungunya, Zika, and, more recently, Oropouche further demonstrate the potential for rapid introduction and expansion of these pathogens in vulnerable territories, where population mobility, high vector density, and lack of prior immunity facilitate their spread^32^ ^,^ ^33^.

Other tropical diseases also pose significant challenges in Colombia. Leishmaniasis, in its various clinical presentations, remains an important public health problem in the tropics, particularly among rural populations affected by deforestation and agricultural expansion, which increase human contact with wild reservoirs and phlebotomine vectors^34^ ^,^ ^35^. Similarly, Chagas disease shows focal transmission primarily in rural tropical areas, where precarious living conditions and inadequate housing create favorable environments for the domiciliation and persistence of triatomine vectors, thereby facilitating pathogen persistence and sustaining local transmission cycles^36^ ^,^ ^37^. Additionally, leptospirosis has re-emerged as a leading bacterial cause of AUFI in several regions of the country, particularly in rural communities frequently affected by recurrent flooding and heavy rainfall, which increase human exposure to soil and water contaminated with the excreta of animal reservoirs^38^ ^,^ ^39^. Despite sustained control efforts, including mass canine vaccination and surveillance campaigns, rabies continues to pose a significant threat in resource-limited tropical regions, where limited prevention programs and inadequate management of domestic dogs and hematophagous bat populations allow ongoing transmission^40^ ^-^ ^42^. Furthermore, the recent re-emergence of yellow fever represents a growing concern in tropical forests and transitional areas, where the virus circulates enzootically among nonhuman primates and mosquito vectors, with the potential to trigger outbreaks among unvaccinated human populations or those with frequent exposure to wildlife^33^ ^,^ ^43^.

Beyond these notifiable diseases, other highly relevant infections in tropical settings remain underdiagnosed, and their importance is often poorly recognized by health professionals and surveillance systems^26^. Rickettsioses, for example, are endemic in several regions and contribute substantially to the burden of AUFI, with high case-fatality rates depending on the infecting species when timely treatment is not provided^44^ ^,^ ^45^. Additionally, emerging and re-emerging diseases, including bartonellosis, hantavirus infections, relapsing fever, and Q fever, remain insufficiently characterized in Colombia. This gap underscores the need for further studies to strengthen knowledge of the epidemiology and public health relevance of these neglected diseases^26^. The inclusion of these agents in clinical practice and epidemiological research is essential for achieving a comprehensive understanding of the country’s tropical disease landscape.

Beyond infectious diseases, injuries caused by venomous animals represent another neglected aspect of tropical medicine in Colombia and other Latin American countries^46^ ^,^ ^47^. Snakebites, scorpion stings, and envenomations by spiders or *Lonomia* spp. caterpillar are the most frequent causes of venom-related injuries, often affecting rural areas and low-income populations^47^ ^-^ ^50^. Despite their clinical relevance and potentially high lethality, these events are often inadequately addressed by health personnel and insufficiently captured by local surveillance systems. This situation highlights the need to strengthen surveillance and medical training to ensure proper diagnosis and management of envenomation cases in tropical settings.

## TROPICAL MEDICINE WITHIN INFECTIOUS DISEASE RESIDENCY PROGRAMS IN COLOMBIA

In Colombia, specialized medical training has evolved in accordance with basic national health priorities, taking into account local epidemiology and the organization of the healthcare system^51^. Within this framework, the subspecialty of infectious diseases aims to train physicians in the diagnosis, management, and prevention of a wide range of infectious diseases, primarily in hospital settings and in response to highly prevalent diseases affecting populations across different regions of the country^52^. Nevertheless, although Colombia encompasses extensive tropical ecosystems and remarkable ecological diversity, tropical medicine has not yet been recognized as a national priority in medical education or established as an independent medical specialty. Therefore, specialized residency programs focused on this field remain absent from current postgraduate training opportunities.

Currently, adult infectious diseases residency programs in Colombia are offered by only a few leading universities located in major cities such as Bogotá, Medellín, and Cali. Only seven institutions offer this program: Pontificia Universidad Javeriana, Universidad de Antioquia, Universidad ICESI, Universidad Nacional de Colombia, Universidad del Rosario, Universidad del Valle, and Universidad Pontificia Bolivariana. These programs provide structured training focused primarily on hospital-based clinical care, including the management of healthcare-associated infections, rational antimicrobial use, and prevalent conditions such as HIV/AIDS, tuberculosis, and respiratory infections. However, tropical medicine is not included as a mandatory training component in all residency programs. Even in infectious diseases residency programs where tropical medicine is formally incorporated into the curriculum, it often represents only a few weeks and limited academic credits within the overall postgraduate training (Table 1). This gap is particularly concerning because tropical regions harbor a broader diversity of infectious agents than any other setting, encompassing virtually all major pathogen groups as well as several geographically restricted ones^53^. Moreover, clinical training in tropical medicine is typically limited to a small number of well-recognized diseases, such as malaria, Chagas disease, leishmaniasis, and dengue, which are highly prevalent in Colombia^28^ ^,^ ^31^ ^,^ ^35^ ^,^ ^36^. Consequently, other clinically relevant conditions, including zoonoses, neglected infections, animal-related envenomations, and malnutrition, remain underrepresented despite their significant contribution to morbidity and mortality in tropical regions of the country^26^ ^,^ ^54^ ^,^ ^55^. Overall, the limited and fragmented inclusion of tropical medicine within infectious diseases residency programs results in insufficient clinical training and may restrict residents’ competence in the diagnosis and management of tropical diseases.

**TABLE 1:** Overview of tropical medicine content in infectious diseases (adults) residency programs in Colombia (1 academic credit equals 48 working hours).

University	Year	Subject	Type	Subject credits	Total program credits	Information website
Universidad Nacional de Colombia	Second	Tropical medicine	Optional	14	129	https://posgrados.unal.edu.co/programa/?id=145
Universidad de Antioquia	Second	Tropical diseases	Core	10	171	https://www.udea.edu.co/wps/portal/udea/web/inicio/unidades-academicas/medicina/estudiar-facultad/posgrados/subespecialidad-enfermedades-infecciosas/Contenido/asMenuLateral/planest
Pontificia Universidad Javeriana	Second	Infectious diseases in tropical medicine	Core	9	133	https://www.javeriana.edu.co/especializacion-infectologia
Universidad del Rosario	First	Tropical medicine	Core	4	103	https://urosario.edu.co/especializacion-en-infectologia
Universidad del Valle	Second	Travel medicine and tropical medicine	Core	4	118	https://salud.univalle.edu.co/la-universidad/rectoria/11-bacteriologia-y-laboratorio-clinico/240-especializacion-en-infectologia
Universidad ICESI	Second	Tropical medicine	Core	5	140	https://www.icesi.edu.co/programas/ciencias-de-la-salud/especializacion-en-infectologia/
Universidad Pontificia Bolivariana	First	Tropical medicine	Core	5	129	https://www.upb.edu.co/es/postgrados/especializacion-infectologia

Additionally, during infectious disease residency training, hospitals where residents complete their clinical rotations are typically located in urban areas. In these settings, tropical medicine cases may occur occasionally, depending on the city and region, but overall exposure is limited because most tropical diseases are concentrated in rural areas, and severe cases are only sporadically encountered in urban hospital-based training settings. Mild and moderately severe cases are also uncommon in these settings, resulting in limited exposure to the full clinical spectrum of tropical medicine^56^. Consequently, many specialists, although well trained in the management of common infections and the rational use of antimicrobials, may have limited experience with tropical diseases and therefore lack the clinical exposure and practical skills required for their accurate diagnosis and management. This gap reflects the low frequency of tropical diseases cases in the major urban hospitals where residency programs are conducted.

Another critical aspect of infectious diseases residency training in Colombia is the limited integration of clinical and research activities related to tropical medicine. Although several institutes and research groups across the country have extensive expertise in this field, infectious diseases residents’ participation in exchanges, fellowships, or collaborative projects with these institutions remains minimal, despite the existence of interinstitutional agreements that facilitate such opportunities. Consequently, limited engagement in tropical disease research reduces opportunities for residents to strengthen scientific skills and competencies in this essential area and may also limit their awareness of the significant public health relevance of these diseases in Colombia.

From a pedagogical perspective, infectious diseases residency programs in Colombia have primarily focused on clinical problem-solving, with no incorporation of courses related to tropical medicine, such as medical entomology, vector ecology, eco-epidemiology of zoonotic diseases, or infectious disease modeling. Thus, infectious diseases residency training in Colombia appears to prioritize the demands of urban hospital-based care while largely overlooking the critical health challenges faced by rural communities, where tropical diseases impose a substantial burden^23^ ^,^ ^57^. Given that Colombia is a tropical country, these educational gaps may exacerbate healthcare inequalities, as tropical diseases predominantly affect peri-urban and rural populations, where specialized expertise is essential but currently insufficient. This situation highlights the urgent need to systematically integrate tropical medicine competencies into residency programs systematically or, ideally, to establish dedicated clinical residency programs in tropical medicine.

## INTERNATIONAL TROPICAL MEDICINE TRAINING PROGRAMS

International experience has demonstrated that tropical medicine constitutes a well-established academic and professional field, supported by specialized training programs that integrate clinical practice, research, and community-based activities in different regions of the world. These programs provide comprehensive training for healthcare professionals to address the complex challenges posed by tropical diseases.

In Europe, the London School of Hygiene and Tropical Medicine has served as a historical benchmark since the early twentieth century^58^ ^-^ ^60^. Its contemporary offering, the Professional Diploma in Tropical Medicine & Hygiene, exemplifies comprehensive training by combining theoretical modules, seminars, laboratory practices, case-based clinical learning, and a substantial fieldwork component in endemic regions, primarily in several African countries. This institution has trained multiple generations of physicians and researchers, many of whom have become internationally recognized experts in clinical management of tropical disease, development of control programs, and epidemic response^58^ ^-^ ^60^.

In the United States, the American Society of Tropical Medicine and Hygiene plays a key role by endorsing the Certificate of Knowledge in Clinical Tropical Medicine and Travelers' Health, which has stimulated the development of specialized training programs in tropical medicine^61^ ^,^ ^62^. Notable examples include the Mayo International Health Program at the Mayo Clinic College of Medicine and Science in Rochester, Minnesota, which focuses on training in tropical diseases within resource-limited and poverty-affected settings^63^. The Diploma Course in Clinical Tropical Medicine and Travelers' Health at Tulane University provides an in-depth comprehensive training in epidemiology, clinical practice, and program planning for disease control^64^. Additionally, the Clinical Tropical Medicine and Traveler´s Health Course at West Virginia University combines intensive hybrid format of online and in-person modules with laboratory and simulation-based training in tropical diseases^65^. Although most of these programs are short-term, they are academically intensive and are distinguished by their interdisciplinary approach, strong practical component, and internationally recognized certification in tropical medicine.

In Latin America, tropical medicine programs are well established primarily in Brazil and Peru, where tropical diseases represent major public health priorities. In Brazil, although undergraduate education specifically focused on tropical and neglected diseases remains limited, instruction in these conditions is incorporated into rural internships and, more intensively, into several excellent postgraduate programs. These programs are offered by leading institutions such as the Federal University of Minas Gerais, University of São Paulo, Oswaldo Cruz Institute (IOC/Fiocruz), Federal University of Pará, State University of Amazonas, among others. These institutions have played central roles in integrating research and clinical training across a wide range of tropical diseases, including leishmaniasis, Chagas disease, schistosomiasis, leprosy, arboviral infections, and emerging zoonoses, while combining teaching, research, and community based-activities^66^ ^-^ ^73^. In Peru, the Alexander von Humboldt Institute of Tropical Medicine at Universidad Peruana Cayetano Heredia offers comprehensive clinical and epidemiological training and serves as a regional reference for tropical medicine^74^. The prestigious Gorgas Course in Tropical Medicine, developed through a collaboration between Universidad Peruana Cayetano Heredia and the University of Alabama at Birmingham, United States, represents one of the most comprehensive and intensive training programs in tropical medicine. The course integrates theoretical instruction, laboratory training, and supervised clinical practice in hospitals and communities located in urban, rural and sylvatic areas of Peru^75^, thereby providing direct exposure to the epidemiological realities of tropical diseases in Latin America.

## FUTURE PERSPECTIVES

International tropical medicine training programs share some standard features within their academic structures that effectively address the diverse challenges posed by tropical diseases, including mandatory public health fieldwork in endemic areas, integration of clinical practice within vulnerable tropical communities, and a strong applied research component. Collectively, these elements reinforce the recognition of tropical medicine as an autonomous and priority medical discipline. 

Unfortunately, in Colombia, these structured clinical training programs in tropical medicine remain absent, despite the country’s extensive tropical ecosystems and the substantial public health burden associated with these diseases. Although some infectious diseases residency programs include short modules on tropical medicine, their duration and scope are insufficient to provide future infectious diseases specialists with comprehensive clinical and practical competencies in this broad medical field.

Although some institutions in Colombia offer postgraduate education in tropical medicine, this training is offered through master's and doctoral programs that are primarily research-oriented. Professionals enrolled in these programs acquire advanced scientific skills and expertise in specific areas of tropical medicine. However, none include clinical components that expose physicians to real cases or provide practical training in the management and treatment of patients affected by these diseases.

To address these gaps, two complementary strategies could be implemented. The first strategy focuses on strengthening tropical medicine training within existing infectious diseases residency programs in Colombia by extending the duration and increasing the number of academic credits assigned to tropical medicine modules, making them a mandatory core component across all institutions offering the program, and establishing partnerships with hospitals and clinical centers located in rural tropical regions to provide field-based clinical experience in tropical diseases. Second, in the longer term, the development of a dedicated clinical postgraduate program in tropical medicine should be considered. Such a program would enable physicians to acquire specialized expertise in the diagnosis, management, and prevention of tropical diseases, while integrating complementary disciplines such as medical entomology and eco-epidemiology. It should also incorporate structured fieldwork in rural vulnerable tropical communities, allowing trainees to gain a comprehensive understanding of the clinical, ecological, and social realities of tropical diseases in the country, thereby promoting more equitable healthcare for populations living in Colombia’s tropical regions. Additionally, these strategies could be complemented by initiatives to encourage early interest in tropical medicine among undergraduate students through faculty-led research groups, thereby fostering the next generation of specialists in this field.

## CONCLUSIONS

Colombia faces a critical gap in clinical training in tropical medicine, despite its geographic location and the substantial public health impact of these diseases. Infectious diseases residency programs, although well-structured for hospital-based care and the management of prevalent infections, allocate limited time to tropical medicine modules, often restricting training to only a few weeks, and in some cases, offering it as an optional module. This limitation reduces future specialists’ clinical competence in managing the wide range of endemic and emerging diseases that primarily affect rural and peri-urban populations. To address this gap, two complementary strategies are proposed: first, strengthening tropical medicine training within existing infectious diseases residency programs; and second, developing a dedicated clinical postgraduate program in tropical medicine to provide physicians with a comprehensive understanding of tropical health. These efforts should be complemented by initiatives that stimulate early interest in tropical medicine among undergraduate medical students. The implementation of these strategies would enhance clinical preparedness, reduce health inequities, strengthen Colombia’s capacity to manage tropical diseases, and improve the country’s readiness to address current and future epidemiological challenges.
